# Association of Body Fat Percentage with Time in Range Generated by Continuous Glucose Monitoring during Continuous Subcutaneous Insulin Infusion Therapy in Type 2 Diabetes

**DOI:** 10.1155/2021/5551216

**Published:** 2021-05-28

**Authors:** Yuting Ruan, Jiana Zhong, Rongping Chen, Zhen Zhang, Dixing Liu, Jia Sun, Hong Chen

**Affiliations:** Department of Endocrinology, Zhujiang Hospital, Southern Medical University, Guangzhou, 510282 Guangdong, China

## Abstract

**Background:**

Obesity is a crucial risk factor associated with type 2 diabetes mellitus (T2DM). Excessive accumulation of body fat may affect the glycemia control in T2DM. This study investigated the relationship between body fat percentage and time in range (TIR) assessed by continuous glucose monitoring (CGM) during short-term continuous subcutaneous insulin infusion (CSII) therapy in T2DM patients.

**Method:**

A total of 85 T2DM patients were recruited in this cross-sectional study. All participants underwent 72 h CGM period during short-term CSII therapy. TIR was defined as the percentage of time spent within the target glucose range of 3.9-10.0 mmol/L. Body composition was measured using bioelectrical impedance analysis (BIA) and overfat was defined as an amount of body fat of at least 25% of total body mass for men or at least 30% for women. Multiple linear regression models were used to evaluate the independent association of body fat percentage with TIR after adjusting for confounding factors.

**Results:**

Compared with normal fat T2DM patients, individual with a higher body fat percentage exhibited lower levels of TIR (*P* = 0.004) and higher 72 h mean blood glucose (72 h MBG) (*P* = 0.001) during short-term CSII treatment. The prevalence of overfat assessed by body fat percentage decreased with the ascending TIR tertiles (*P* < 0.05). Multiple linear regression analysis indicated that body fat percentage was significantly associated with TIR independent of age, gender, diabetes duration, HbA1c, and BMI (*P* = 0.043).

**Conclusions:**

Body fat percentage was significantly associated with TIR in T2DM during short-term CSII therapy. Reduction of body fat may be an important therapeutic target to improve glycemic control in high body fat T2DM patients, who may benefit less from intensive insulin treatment.

## 1. Introduction

With the rapid economic development, population aging, sedentary lifestyles and excessive eating, the prevalence of both type 2 diabetes mellitus (T2DM) and obesity is rapidly increasing worldwide, reaching pandemic levels. The 2017 International Diabetes Federation (IDF) report showed that the number of diabetic patients in China reached 114.4 million in 2017 [1]. The prevalence of obesity increased from 3.2% to 10.8% in adult men and from 6.4% to 14.9% in adult women, reaching nearly 90 million in China, making it the country with the largest number of obese people in the world [2]. Compared with normal weight individual, obesity were nearly five times more likely to develop T2DM [3], as well as increased the difficulty of glycemia control. Besides, a recent study indicated that high BMI or waist circumference was significantly associated with increased glycemia in nondiabetic population, suggesting poor glycemia control in obese patients [4]. Even though BMI or waist circumference is used as an indicator of obesity, they cannot reflect the quantity and distribution of body fat, existing some limitations in assessing body composition. Recently, bioelectrical impedance analysis (BIA) has been developed to measure body composition, which has been shown to be a more convenient, practical, and less invasive method for evaluating body fat [5]. A retrospective cross-sectional study showed that body fat percentage, rather than BMI, was related to the risk of T2DM, indicating that the accumulation of body fat might closely correlated with the glycemia control [1]. Despite the use of body fat percentage in assessing body composition, the relation between body fat percentage and glycemic control in T2DM remains unknown.

Hyperglycemia may impair *β*-cells and decrease insulin sensitivity, which were crucial pathophysiological defects in T2DM [6]. Continuous subcutaneous insulin infusion (CSII) as an important transient aggressive glycemic control method to protect *β*-cells and induce normoglycemia accompanied with the restoration of insulin sensitivity has been used in T2DM patients with severe hyperglycemia [7]. Weng et al. [8] also reported that CSII in T2DM patients has more favourable outcomes on recovery of *β*-cell function and shortens the period of antecedent glucotoxicity and protracted drug-free remission compared with patients in oral hypoglycaemic agent groups. Thus, the potential benefits of intensive intervention with CSII to counter both insulin resistance and *β*-cell dysfunction in T2DM patients with severe hyperglycemia should be taken into consideration.

The 2020 American Diabetes Association (ADA) guidelines recommend that patients using intensive insulin therapy should be encouraged to assess glucose levels by continuous glucose monitoring (CGM) [9]. CGM continuously captures the glucose profile over a number of days and may be the optimal way to identify an individual's current glycemic status [10]. Time in range (TIR) assessed by CGM refers to the time an individual spends within the target glucose range of 3.9–10.0 mmol/L during a 24 h period, which provides valuable information about whether the frequency and duration of hypoglycemia or hyperglycemia improve over time. In addition, TIR measurements are useful for evaluating and comparing the response of T2DM to insulin during short-term CSII therapy. In recent years, it has been reported that TIR was associated with both macrovascular disease [11] and diabetes microvascular complications, such as diabetic retinopathy [12], the development of microalbuminuria [13], and peripheral nerve function [14]. However, despite increased TIR interest and attention, there is little insight into the relationship between body composition and TIR during short-term CSII treatment, which might provide us with more insights for disease management. Therefore, the current study is aimed at investigating the association of body fat percentage with TIR obtained from CGM in patients with T2DM during short-term CSII therapy.

## 2. Research Design and Method

### 2.1. Study Population

This cross-sectional observational study was conducted in T2DM patients who had received continuous subcutaneous insulin infusion therapy and were monitored by a CGM system at the inpatient department of Zhujiang Hospital of Southern Medical University from July 2019 to October 2020. A total of 85 patients with T2DM according to WHO criteria (1999) [15] were consecutively recruited. The patients, aged ≥18 years, had the levels of HbA1c ≥ 8.0%. Exclusion criteria included type 1 diabetes; pregnancy or lactation; acute diabetic complications, such as diabetic ketoacidosis; use of medications or drugs that may influence glucose metabolism (i.e., steroids and thiazide diuretics); and a history of mental disorders, thyroid function disorders, or severe kidney or liver dysfunction. This study protocol was approved by the ethics committees of Zhujiang Hospital of Southern Medical University in accordance with the principles of the Declaration of Helsinki.

### 2.2. Anthropometric and Biochemical Measurements

Each patient underwent a physical examination that included measurements of body weight, height, waist circumference, hip circumference and systolic blood pressure (SBP), and diastolic blood pressure (DBP). The BMI was calculated as weight in kilograms divided by height in meters squared (kg/m^2^). Waist-to-hip ratio (WHR) is calculated as waist circumference (cm) divided by hip circumference (cm). Blood pressure was measured three times, and the average of three recordings was calculated for further analysis. Patients with a history of hypertension or abnormally high arterial blood pressure (SBP ≥ 140 mmHg or DBP ≥ 90 mmHg) were considered as hypertension. Fatty liver disease was evaluated by liver doppler ultrasound examination. Smoking was defined as daily cigarette use for at least 12 months, regardless of the amount.

Venous blood samples were drawn from the antecubital vein after an overnight fast of 8-10 h. The blood analyses were conducted in the laboratory of Zhujiang Hospital, Southern Medical University, for the measurement of biochemical markers, such as blood glucose, glycated hemoglobin (HbA1c), C-peptide, lipid profile including triglycerides (TG), total cholesterol (TC), high-density lipoprotein cholesterol (HDL-C), and low-density lipoprotein cholesterol (LDL-C) and uric acid (UA).

### 2.3. Continuous Subcutaneous Insulin Infusion (CSII) Therapy

All participants were treated with short-term intensive insulin therapy using insulin pumps (Paradigm 712 pump, Medtronic Inc., Northridge, CA) with Insulin Aspart (Novo Nordisk, Bagsværd, Denmark) for at least three consecutive days. The initial insulin doses were 0.4-0.5 IU/kg, and total daily doses were divided into 50% of basal and 50% of premeal. Dosages were titrated every day based on the fasting and postprandial glucose by an experienced physician in order to achieve glycemic goal. Glycemic targets were defined as fasting/premeal blood glucose less than 6.1 mmol/L and 2 h postprandial blood glucose less than 8.0 mmol/L. No other hypoglycemic agents were added during short-term CSII therapy.

### 2.4. CGM Parameters

All participants were monitored with a CGM system FreeStyle Libre Pro 1.0 (Abbott Inc., USA) as soon as they receive CSII therapy. The sensor of the CGM system was inserted on the first day and removed after 72 h, generating a total of 288 consecutive sensor value records. After the 3-day monitoring period, the glucose profiles were downloaded from the CGM system; then, the TIR and other glycemic metrics were calculated based on the original glucose data recorded by this system. TIR was defined as the percentage of time spent within the target glucose range of 3.9-10.0 mmol/L during a 24 h period, while time above range (TAR) was above the target glucose (>10.0 mmol/L), and time below range (TBR) was below the target glucose (<3.9 mmol/L). Besides, a series of parameters concerning 72 h mean blood glucose (72 h MBG) and glycemic variability metrics, including standard deviation of glucose (SD), coefficient of variation (CV), mean amplitude of glycemic excursions (MAGE), and mean of daily differences (MODD) were detected [16]. Among them, MAGE was used to evaluate the intraday glycaemia variability by calculating the arithmetic mean of the differences between consecutive peaks and nadirs, and only excursions of more than one SD of the mean glycemic value were considered [17]. MODD, as the average absolute difference of paired sensor glucose values during two successive days, was used to assess the day-to-day glycemic variability [18].

### 2.5. Body Composition Analysis

Body composition was measured using Jawon bioelectrical impedance analyzer (BIA) ioi353 (Jawon Medical Co., Ltd., Korea), which processes 15 reactance measurements using tetrapolar 8-point tactile electrode system at three different frequencies (5, 50, 250 KHZ) at each of five segments of the body (right arm, left arm, right leg, left leg, and trunk), so as to estimate the body fat percentage, muscle quantity, and visceral fat area. Before the examination, the participants were required to empty their bladder, wear light clothes, expose their limbs for electrode attachment, and rest for 5 min to ensure that accurate measurements were captured. The body composition analysis was carried out within 1 week of the intensive insulin therapy. This widely used measurement is safe, noninvasive, highly precise with comprehensive indicators, and easy to carry out in clinical settings [19, 20]. The enrolled patients were grouped into overfat and normal fat according to the body fat percentage. Overfat was defined as an amount of body fat of at least 25% of total body mass for men and at least 30% for women [21, 22].

### 2.6. Statistical Analyses

All statistical analyses were conducted using SPSS 26.0 software (IBM Corporation, Armonk, NY, USA). Normally distributed data are expressed as the mean ± SD, whereas skewed clinical data are expressed as median value (interquartile range). Categorical variables are expressed as frequencies (percentages). Student's *t* test or Mann-Whitney *U* tests was used for continuous variables, as appropriate. The chi-square test was carried out for categorical data. The TIRs were divided into three groups with the tertiles as the cut point. The normally distributed multiple samples were assessed using one-way ANOVA, and Jonckheere-Terpstra test was used for nonnormally distributed data. Multiple linear regression analysis was performed to assess the independent association of body fat percentage with TIR after controlling for covariates including age, gender, diabetes duration, HbA1c, and BMI. A value of *P* < 0.05 (two-sided) was considered statistically significant.

## 3. Results

### 3.1. Baseline Characteristics of Study Population

**Figure 1:**
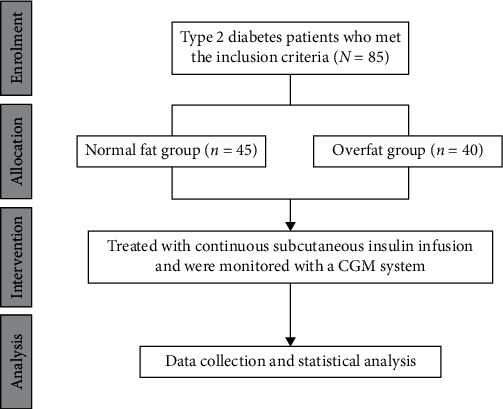
Flow diagram of the study design.

The 85 enrolled T2DM patients were grouped into overfat (*n* = 40) and normal fat (*n* = 45) according to the body fat percentage. All subjects demonstrated high levels of HbA1c ≥ 8.0%, and all of them had received short-term CSII therapy and were monitored by a CGM system. The flow diagram of the study design was shown in Figure 1. The clinical characteristics of the patients grouped by body fat percentage were summarized in Table 1. Overall, the mean age of all subjects was 57.6 ± 10.7 years, the median duration of diabetes was 72 months, and they had a mean ± SD HbA1c of 11.0 ± 1.8%. Patients in overfat group shown a higher level of BMI, waist-to-hip ratio, body fat quantity, and visceral fat area (all *P* for trend <0.05). Furthermore, TIR, 72 h MBG and CV also differed statistically across the various groups (all *P* < 0.05), while the difference could not be observed in HbA1c or diabetes duration. TIR was 54.3 ± 17.6% in the normal fat group and 41.8 ± 20.8% in the overfat group (*P* = 0.004). Among the glucose values out of the range of 3.9-10.0 mmol/L, TAR was substantially higher in overfat patients compared with those with normal body fat (*P* = 0.001), while TBR was lower in overfat than that of normal fat ones (*P* = 0.003). Besides, the overfat participants were more likely to suffer from hypertension and fatty liver disease (*P* < 0.05 for both).

### 3.2. Characteristics of Study Participants by Tertiles of TIR

Next, all of the participants were stratified into groups according to tertiles of the TIR (Tertile 1 [T1]: ≤43.75%; Tertile 2 [T2]: 43.76-57.29%; Tertile 3 [T3]: ≥57.30%).  Table 2 depicts the characteristics of patients by TIR tertiles. Significant differences were detected in TIR and glycemic variability measures including 72 h MBG, SDBG, CV, and MODD among the TIR tertiles (all *P* for trend <0.05), whereas the body composition indicators, such as BMI, waist circumference, and body fat percentage, did not reach statistical differences between groups. Participants with the highest tertiles of TIR (T3) exhibited lower levels of triglycerides (*P* = 0.008).

### 3.3. Prevalence of Obese Patients Stratified by Body Fat Percentage, BMI, Waist Circumference, or Visceral Fat Area

**Figure 2:**
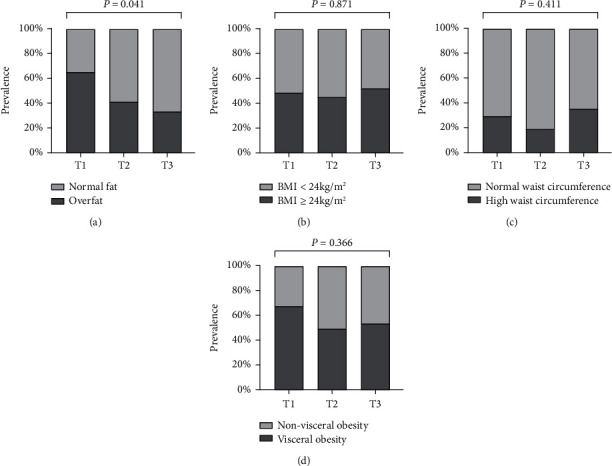
Prevalence of obese participants stratified by body fat percentage, BMI, waist circumference, or visceral fat area among different tertiles of TIR^a^. (a) Prevalence of “normal fat” and “overfat” in different tertiles (T1-T3) of TIR. Overfat was defined as an amount of body fat of at least 25% of total body mass for men and at least 30% for women. The prevalence of overfat was 65.5% in T1 group, 41.4% in T2 group, and 33.3% in T3 group (*P* = 0.041). *P* value for the significant difference among the groups was determined by *χ*^2^ test. (b) Prevalence of “BMI < 24 kg/m^2^” and “BMI ≥ 24 kg/m^2^” in different tertiles (T1-T3) of TIR. The prevalence of “BMI ≥ 24 kg/m^2^” was 48.3% in T1 group, 44.8% in T2 group, and 51.9% in T3 group (*P* = 0.871). *P* value for the significant difference among the groups was determined by *χ*^2^ test. (c) Prevalence of “normal waist circumference” and “high waist circumference” in different tertiles (T1-T3) of TIR. A waist circumference > 88 cm in women and >102 cm in men was classified as high waist circumference. The prevalence of high waist circumference was 28.6% in T1 group, 18.5% in T2 group, and 34.6% in T3 group (*P* = 0.411). *P* value for the significant difference among the groups was determined by *χ*^2^ test. (d) Prevalence of “nonvisceral obesity” and “visceral obesity” in different tertiles (T1-T3) of TIR. The visceral obesity was defined as visceral fat area ≥ 100 cm^2^. The prevalence of visceral obesity was 67.9% in T1 group, 50.0% in T2 group, and 53.8% in T3 group (*P* = 0.366). *P* value for the significant difference among the groups was determined by *χ*^2^ test. ^a^TIR T1 ≤ 43.75%, T2: 43.76-57.29%, T3: ≥57.30%.

Considering the cut-off value of body fat percentage is different in gender, we thus further compared the proportion of overfat patients defined by body fat percentage stratified by sex in different TIR tertiles groups. In general, with the ascending tertiles of TIR, the prevalence of overfat assessed by body fat percentage decreased (*P* = 0.041) (Figure 2(a)). However, there were no such trends in the categories of BMI, waist circumference or visceral fat area (all *P* > 0.05), suggesting body fat percentage may be a strong predictor of glycemic control in T2DM patients during CSII therapy (Figures 2(b)–2(d)).

### 3.4. Relationship between Body Fat Percentage and TIR

A multiple linear regression analysis was performed to evaluate the relationship between body fat percentage and TIR. As described in Table 3, strong relationship existed between body fat percentage and TIR after adjusting for confounding factors, including age, gender, diabetes duration, HbA1c, and BMI (*β* = −0.435, *P* = 0.043).

## 4. Discussion

Obesity is known to have an insidious onset and predisposes toward several metabolic disturbances that threaten human health. Excessive ectopic accumulation of adipose tissue in the body and changes in body composition are crucial factors in the development of obesity-related insulin resistance and T2DM [23], as well as affect the glycemic control. A retrospective cohort study indicated that baseline BMI is one of the most accurate predictors of the future glycemic control in T2DM patients [24]. Previous cross-sectional study has also showed that HbA1c was significant and positive associated with increased waist circumference in T2DM participants [25]. However, the parameter of BMI or waist circumference to assess obesity-related complications is not enough. In this study, we elaborated on the correlation between body composition, assessed by bioelectrical impedance analyzer (BIA), and TIR assessed by CGM in T2DM patients during short-term continuous subcutaneous insulin infusion (CSII) therapy. Our findings showed that body composition, particularly high body fat percentage, may contribute to decreased TIR in obese T2DM population. To the best of our knowledge, this was the first study to evaluate the association between body composition and CGM-assessed TIR especially during short-term intensive insulin therapy in Chinese obese T2DM patients.

As a convenient, practical, and less invasive method to assess body composition, BIA was widely used in clinical practice. A cross-sectional study has demonstrated that total body fat mass assessed by BIA were strongly associated with insulin resistance in T2DM [26]. Besides, Hameed et al. studied the impact of visceral fat in T2DM and found that visceral adiposity index was positively associated with the presence of T2DM and had a significant negative outcome over glycemic control [27, 28]. Since ectopic fat accumulation was significantly related to body fat percentage measured by BIA in a previous study [29], it is possible that body fat percentage can reflect ectopic fat quantity and may become an optimal predictor of T2DM glycemia management. Currently, the results of our study showed that body fat percentage was significantly and independently correlated with glycemia control. We also found a poor controlled glycemia in T2DM patients with relatively high body fat percentage during intensive hypoglycemic therapy, suggesting the accumulation of body fat may be the main cause of the poor glycemia control in obese T2DM populations.

Continuous subcutaneous insulin infusion (CSII) therapy has been of value in T2DM patients who fail to achieve adequate glycemic control [30]. More physiological delivery of insulin by continuous subcutaneous infusion has been proven for reduction of glucose toxicity and prevention of progressive *β*-cell dysfunction in T2DM [31]. Numerous studies have demonstrated that excellent glycemic control can be achieved without need for further medication after short-term CSII therapy in T2DM population [32–34]. However, the different characteristics in T2DM patients, such as the degree of obesity, may affect the time to euglycaemia during intensive interventions [8]. Similarly, in our study, the efficacy of glycemic control during transient CSII treatment varies considerably among patients with different body fat percentage. It would be presumed that those with more body fat accumulation might have more severe insulin resistance but, perhaps, the less glucose response. Therefore, short-term CSII treatment may not be the best intensive intervention for high body fat T2DM individuals with severe hyperglycemia, while hypoglycemic agents combined with body fat reduction might be the better choices in clinical practice.

With advances in CGM technology, time in range (TIR) of 3.9-10 mmol/L has been introduced by the 2020 ADA guidelines as an intuitive and key parameter of short-term glycemic management [9]. A series of previous studies have also shown that TIR not only could be used to assess the risk of microvascular complications [12, 14, 35], but also predict the all-cause mortality from cardiovascular events in T2DM [11], further supporting TIR as an acceptable glucose metric as well as a reasonable end point for clinical trials. In the current study, with ascending tertiles of TIR, the percentage of overfat patients classified by body fat percentage decreased as compared to other grouping methods (such as BMI, waist circumference, or visceral fat area), suggesting body fat percentage may had a more significant negative outcome over glycemic control during CSII therapy. Recently, a study demonstrated that regardless of mean glucose, HbA1c, or glycemic variability metrics had an impact on TIR [36, 37]. Based on the previous study, we further found a robust correlation between body fat percentage and TIR. The effect of body fat percentage on TIR was independent of age, gender, HbA1c, diabetes duration, and BMI. Thus, our study provides evidence of an independent effect of body fat percentage on TIR, indicating that quantitative assessment of body fat and further considering body fat percentage as part of management strategy are fundamentally important.

Furthermore, glycemic variability was also taken into consideration in our study when evaluating quality of glycemic control during short-term CSII therapy in T2DM. A previous cross-sectional study reported that subjects with a higher BMI or waist circumference had higher levels of 72 h MBG assessed by CGM system [4]. Our study was in consistent with the result of previous study, demonstrating higher levels of 72 h MBG in obese T2DM patients than that of nonobese ones during CSII therapy. However, the decrease of glycemic variability CV was observed in patients with high body fat percentage. Among glycemic variability parameters, CV was significantly correlated with the risk of hypoglycemia [38]. In the current study, TBR, defined as the percentage of time spent below the target glucose range (<3.9 mmol/L), was lower in participants with higher body fat percentage, indicating the risk of hypoglycemic was relatively decreased during CSII therapy in obese subjects. A previous study reported that obese patients exhibited a little bit better pancreatic *β*-cell function in comparison with that observed in the nonobese subjects [39], which may contribute to decreased glucose fluctuation in T2DM individual with relatively high body fat percentage. It is notable that clinicians should pay more attention to the increased risk of hypoglycemia during intensive hypoglycemic treatment in lean T2DM patients.

Several limitations of this study should be addressed. First, this was a cross-sectional study, and thus, we could not examine the causal relationship between body composition and TIR. Besides, considering the small overall sample size in our study, the results be replicated in larger study populations is warranted. Finally, we estimated body composition based on the BIA, not by the “gold standard” method, such as computer tomography (CT) and magnetic resonance imaging (MRI); however, CT or MRI is expensive and not easily feasible in a relatively large-scale study, and we believe that proxy measures are reliable according to the previous studies [40].

## 5. Conclusions

High body fat percentage adversely affect the glycemic control in T2DM individual during continuous subcutaneous insulin infusion therapy. Reduction of body fat may be crucial therapeutic target in improving glycemic control in high body fat T2DM patients.

## Figures and Tables

**Table 1:** Characteristics of study participants by body fat percentage.

Variables	All subjects (*n* = 85)	Normal fat group (*n* = 45)	Overfat group (*n* = 40)	*P* value
Age (years)	57.6 ± 10.7	55.9 ± 9.8	59.4 ± 11.4	0.132
Male (*n*, %)	54 (63.5%)	34 (75.6%)	20 (50.0%)	0.018
Diabetes duration (months)	72.0 (2.5-138.0)	60.0 (2.5-120.0)	78.0 (2.3-144.0)	0.684
Insulin doses (IU/kg/d)	0.50 ± 0.1	0.49 ± 0.1	0.52 ± 0.1	0.307
Bolus/basal ratio	1.21 ± 0.2	1.23 ± 0.2	1.19 ± 0.1	0.269
SBP (mmHg)	131.5 ± 18.7	128.5 ± 19.2	135.0 ± 17.7	0.112
DBP (mmHg)	77.2 ± 10.9	76.5 ± 9.6	78.0 ± 12.3	0.528
BMI (kg/m^2^)	24.0 ± 2.9	22.4 ± 2.2	25.7 ± 2.7	<0.001
Waist circumference (cm)	92.3 ± 10.9	87.8 ± 7.7	96.9 ± 11.8	<0.001
Hip circumference (cm)	94.7 ± 6.5	92.5 ± 7.0	97.0 ± 5.2	0.002
Waist-to-hip ratio (WHR)	0.97 ± 0.1	0.95 ± 0.1	1.00 ± 0.1	0.026
TC (mmol/L)	5.0 (4.2-5.9)	4.8 (3.9-5.4)	5.3 (4.3-6.1)	0.066
TG (mmol/L)	1.8 (1.2-3.0)	1.5 (1.1-3.0)	1.9 (1.3-3.2)	0.297
HDL-C (mmol/L)	1.0 ± 0.2	1.0 ± 0.3	1.0 ± 0.2	0.990
LDL-C (mmol/L)	3.1 ± 1.1	3.0 ± 1.0	3.3 ± 1.2	0.191
HbA1c (%)	11.0 ± 1.8	10.8 ± 1.8	11.2 ± 1.7	0.305
Fasting C-peptide (ng/mL)	1.6 (1.2-2.8)	1.4 (1.1-2.3)	2.0 (1.2-3.3)	0.101
Postprandial C-peptide (ng/mL)	2.8 (1.9-4.7)	2.6 (1.8-4.5)	2.9 (2.2-5.3)	0.127
72 h MBG (mmol/L)	10.4 ± 2.3	9.6 ± 2.1	11.3 ± 2.2	0.001
SDBG (mmol/L)	3.3 (2.8-3.9)	3.3 (2.7-3.8)	3.4 (2.8-4.1)	0.489
CV (%)	34.2 ± 7.7	35.9 ± 7.6	32.3 ± 7.6	0.033
MAGE (mmol/L)	4.6 ± 1.3	4.4 ± 1.2	4.8 ± 1.3	0.139
MODD (mmol/L)	3.3 (2.4-3.9)	3.0 (2.2-3.8)	3.5 (2.5-4.2)	0.109
TIR (%)	48.4 ± 20.1	54.3 ± 17.6	41.8 ± 20.8	0.004
TAR (%)	49.0 ± 22.5	41.7 ± 20.9	57.4 ± 21.5	0.001
TBR (%)	0 (0-1.7)	0.4 (0-8.0)	0 (0-0.9)	0.003
Muscle quantity (kg)	44.0 ± 8.0	44.9 ± 7.5	43.1 ± 8.4	0.305
Body fat quantity (kg)	16.7 ± 5.0	13.5 ± 3.6	20.3 ± 3.6	<0.001
Body fat percentage	25.8 ± 6.3	21.8 ± 5.1	30.4 ± 4.0	<0.001
Visceral fat area (cm^2^)	101.8 ± 37.0	88.0 ± 30.8	117.7 ± 37.6	<0.001
Hypertension (*n*, %)	38 (44.7%)	13 (28.9%)	25 (62.5%)	0.002
Fatty liver (*n*, %)	55 (64.7%)	24 (55.8%)	31 (83.8%)	0.007
Current smoker (*n*, %)	22 (25.9%)	15 (34.1%)	7 (17.5%)	0.084

Data are represented as mean ± SD, median [25th to 75th percentile range], or number (%). DBP: diastolic blood pressure; SBP: systolic blood pressure; BMI: body mass index; TC: total cholesterol; TG: triglycerides; HDL-C: high density lipoprotein cholesterol; LDL-C: low density lipoprotein cholesterol; 72 h MBG: 72 h mean blood glucose concentration; SDBG: standard deviation of blood glucose; CV: coefficient of variation; MAGE: mean amplitude of glycemic excursions; MODD: mean of daily differences; TIR: time in range; TAR: time above range; TBR: time below range.

**Table 2:** Characteristics of patients according to the TIR tertiles.

Variables	TIR tertiles			
	T1 (≤43.75%) (*n* = 29)	T2 (43.76-57.29%) (*n* = 29)	T3 (≥57.30%) (*n* = 27)	*P* value
Age (years)	58.6 ± 10.1	55.5 ± 11.7	58.7 ± 10.2	0.428
Male (*n*, %)	18 (62.1%)	20 (69.0%)	16 (59.3%)	0.737
Diabetes duration (months)	96.0 (4.0-126.0)	48.0 (1.0-138.0)	72.0 (4.0-180.0)	0.702
Insulin dosage (IU/kg/d)	0.55 ± 0.1	0.50 ± 0.1	0.45 ± 0.1	0.006
SBP (mmHg)	134.2 ± 18.1	131.2 ± 19.7	129.0 ± 18.6	0.578
DBP (mmHg)	76.7 ± 10.3	79.2 ± 10.5	75.6 ± 12.0	0.453
BMI (kg/m^2^)	24.0 ± 2.4	23.8 ± 3.7	24.1 ± 2.6	0.949
Waist circumference (cm)	94.3 ± 12.6	91.1 ± 10.6	91.4 ± 9.1	0.493
Hip circumference (cm)	94.5 ± 4.5	94.0 ± 9.0	95.7 ± 5.4	0.591
Waist-to-hip ratio (WHR)	1.00 ± 0.1	0.97 ± 0.1	0.95 ± 0.1	0.237
TC (mmol/L)	5.1 (4.4-6.2)	5.0 (4.3-5.9)	4.6 (3.8-5.6)	0.058
TG (mmol/L)	2.1 (1.3-3.4)	2.1 (1.3-3.8)	1.2 (0.8-2.0)	0.008
HDL-C (mmol/L)	1.0 ± 0.2	1.0 ± 0.3	1.0 ± 0.3	0.374
LDL-C (mmol/L)	3.3 ± 1.2	3.1 ± 1.0	2.9 ± 1.1	0.386
HbA1c (%)	11.6 ± 1.7	10.9 ± 1.2	10.6 ± 2.2	0.080
Fasting C-peptide (ng/mL)	1.9 (1.2-3.0)	1.4 (0.9-2.2)	1.5 (1.1-3.1)	0.495
Postprandial C-peptide (ng/mL)	2.8 (2.2-4.9)	2.7 (1.6-5.0)	3.5 (1.9-4.7)	0.994
TIR (%)	26.6 ± 13.1	49.8 ± 3.7	70.4 ± 8.3	<0.001
72 h MBG (mmol/L)	12.8 ± 1.5	10.2 ± 1.0	8.1 ± 1.1	<0.001
SDBG (mmol/L)	3.8 (3.2-4.8)	3.5 (3.1-4.0)	2.7 (2.5-3.4)	<0.001
CV (%)	30.9 ± 7.5	36.2 ± 8.5	35.7 ± 5.9	0.014
MAGE (mmol/L)	4.9 ± 1.3	4.5 ± 1.4	4.3 ± 1.1	0.178
MODD (mmol/L)	3.5 (2.7-4.1)	3.5 (2.4-4.6)	3.0 (1.9-3.4)	0.017
Muscle quantity (kg)	43.2 ± 8.4	44.6 ± 9.0	44.4 ± 6.4	0.772
Body fat quantity (kg)	17.5 ± 3.1	16.1 ± 6.9	16.6 ± 4.1	0.498
Body fat percentage	27.5 ± 5.4	24.3 ± 7.3	25.7 ± 5.8	0.149
Visceral fat area (cm^2^)	111.8 ± 33.3	96.4 ± 44.7	96.8 ± 30.2	0.180
Hypertension (*n*, %)	15 (51.7%)	12 (41.4%)	11 (40.7%)	0.644
Fatty liver (*n*, %)	20 (74.1%)	18 (66.7%)	17 (65.4%)	0.760
Current smoker (*n*, %)	7 (25.0%)	7 (24.1%)	8 (29.6%)	0.883

Data are represented as mean ± SD, median [25th to 75th percentile range], or number (%). DBP: diastolic blood pressure; SBP: systolic blood pressure; BMI: body mass index; TC: total cholesterol; TG: triglycerides; HDL-C: high density lipoprotein cholesterol; LDL-C: low density lipoprotein cholesterol; 72 h MBG: 72 h mean blood glucose concentration; SDBG: standard deviation of blood glucose; CV: coefficient of variation; MAGE: mean amplitude of glycemic excursions; MODD: mean of daily differences; TIR: time in range.

**Table 3:** Multiple linear regression analyses of TIR (dependent variable).

Variable	SE	Standardized *β*	*P* value
Age	0.002	0.069	0.566
Gender	0.075	0.279	0.128
HbA1c	0.013	-0.205	0.073
Body fat percentage	0.007	-0.435	0.043
Diabetes duration	0.000	-0.178	0.142
BMI	0.011	0.184	0.250

TIR: time in range. TIR was regarded as the dependent variable, and independent variables included age, gender, HbA1c, diabetes duration, BMI, and body fat percentage. *β*: regression coefficient.

## Data Availability

The datasets used and analyzed during the current study are available from the corresponding author.

## Abbreviations

T2DM: Type 2 diabetes mellitus
BMI: Body mass index
BIA: Bioelectrical impedance analysis
CGM: Continuous glucose monitoring
TIR: Time in range
TAR: Time above range
TBR: Time below range
72 h MBG: 72 h mean blood glucose
SDBG: Standard deviation of blood glucose
CV: Coefficient of variation
MAGE: Mean amplitude of glycemic excursions
MODD: Mean of daily differences
CSII: Continuous subcutaneous insulin infusion.
